# Circulating microRNA-375 as biomarker of pancreatic beta cell death and protection of beta cell mass by cytoprotective compounds

**DOI:** 10.1371/journal.pone.0186480

**Published:** 2017-10-17

**Authors:** Imane Song, Sarah Roels, Geert A. Martens, Luc Bouwens

**Affiliations:** 1 Cell Differentiation Lab, Vrije Universiteit Brussel (VUB), Brussels, Belgium; 2 Diabetes Research Center, Vrije Universiteit Brussel (VUB), Brussels, Belgium; Baylor College of Medicine, UNITED STATES

## Abstract

**Objective:**

Previous studies demonstrated that circulating microRNA-375 (miR-375) is a suitable plasma biomarker for real-time detection of beta cell death. The present study evaluated the use of this biomarker to assess the beta cytoprotective effect of phenylpropenoic acid glucoside (PPAG), which was previously demonstrated to protect beta cells against various types of injury, and of exendin-4, which is an established antidiabetic drug.

**Methods:**

PPAG or exendin-4 were administered in mice treated with streptozotocin (STZ) to acutely induce beta cell death. Beta cell mass and apoptotic death were measured in pancreatic tissue sections. Circulating miR-375 was measured in blood plasma by RT-qPCR. The release of miR-375 was also measured in vitro by MIN-6 beta cells.

**Results:**

Administration of STZ resulted in measurable circulating levels of miR-375, a decrease in beta cell mass and increase in frequency of apoptotic beta cells. In vitro, there was a good correlation between miR-375 release and the extent of beta cell death. Treatment of mice with PPAG or exendin-4 significantly attenuated STZ-induced loss of beta cell mass and beta cell apoptosis, and normalized the blood level of miR-375.

**Conclusions:**

These findings show the potential use of serological miR-375 measurements to evaluate the beta cytoprotective effect of (potential) antidiabetic drugs in vivo.

## 1. Introduction

Type 2 diabetes and prediabetes are two of the top pressing health issues worldwide and there is an important unmet need in new medication to prevent or halt the disease.

Besides dysregulated blood sugar levels and the devastating effects of chronic hyperglycemia, diabetes is characterized by progressive failure and loss of pancreatic beta cells [1–4]. Preservation or restoration of the insulin-producing beta cell mass is therefore an important target for new drug development [5–8].

Normal postnatal maintenance or growth of the pancreatic beta cell mass is considered to result exclusively from mitotic division of existing beta cells [9]. However, this mechanism can be counterbalanced or outweighed by beta cell loss resulting from cell death. Therefore, restoration or preservation of beta cell mass could be achieved by drugs acting on beta cell replication and/or beta cell death. We reported that oral administration of a phenylpropenoic acid glucoside (PPAG), originally discovered as a phytochemical from the medicinal Rooibos plant, prevented the development of diabetes in mice fed a high fat diet [10]. PPAG has also been attributed a glucose-lowering effect [11] and our study demonstrated a direct beta cell protective effect in vivo and in vitro [10,12]. Streptozotocin (STZ) treatment of mice represents a convenient model for rapid pharmacological screening of potential antidiabetic, beta cell protecting drugs. In this short-term model, changes in beta cell mass and apoptotic index that are caused by injury or by cytoprotective compounds can be measured with immunohistochemical methods in pancreatic tissue. However, to potentially demonstrate beta cytoprotective effects in new treatments of (pre-)diabetics, non-invasive methods are needed. Measuring changes in the beta cell mass by imaging methods like positron emission tomography (PET) is a key tool for optimizing diabetes prevention and treatment but is still in its infancy due to lack of highly specific and sensitive beta cell tracers as well as technical limitations of the imaging tools [13].

Another promising issue is the use of serological biomarkers reflecting beta cell death or damage that are released in the circulation. MicroRNAs (miRNAs) are interesting candidates for this due to their stability in the circulation [14] and their sensitive detection by polymerase chain reaction (PCR).

MicroRNAs are short non-coding RNA molecules of about 22 nucleotides long that function as regulators of gene expression. Their presence in the circulation is attributed primarily to leakage from dead or damaged cells. Extracellular microRNAs circulate in blood mostly as part of 96 kDa macromolecular Argonaute complexes, that shield the microRNAs from plasma RNAse, thus conferring high stability and extended circulation times [15].

MicroRNA-375 (miR-375) has been demonstrated to represent an islet-enriched miRNA that is highly expressed in pancreatic islets of humans and mice and is required for proper beta cell functioning as well as maintaining a normal beta cell mass [16].

Erener et al. [17] showed that miR-375 is a suitable blood marker to detect beta cell death and predict diabetes in STZ-treated and NOD mice. Other studies have shown its usefulness in detecting beta cell damage in type 1 diabetes or following clinical islet transplantation in order to evaluate different anti-inflammatory protocols [18,19].

In the present study, we used miR-375 to assess the beta cytoprotective effect of PPAG in an acute model of beta cell damage induced by a single injection of STZ [12]. Serological levels of miR-375 were compared to histological measurements of beta cell mass and beta cell apoptosis. PPAG was also compared to exendin-4, an established antidiabetic and beta cytoprotective drug.

## 2. Materials and methods

### 2.1. In vitro

MIN-6 beta cell line (a kind gift from prof. H. Heimberg, Beta cell neogenesis, Brussels) was cultured in DMEM medium supplemented with 10% FCS. The cells were incubated with different concentrations of streptozotocin (Sigma Aldrich), ranging from 0–10 mM, for 18 hours. Culture supernatant was collected from each condition and subjected to miR-375 analyses to evaluate miR-375 discharge. Cells were stained using crystal violet to determine beta cytotoxicity. Absorbance was measured at 540 nm with a microplate reader. Experiments were conducted in triplicate and repeated three times. Percentage cytotoxicity was calculated using following equation:

$$\left(\frac{O.D_{Vehicle}\;-O.D_{STZ}}{O.D_{Vehicle}}\right)\times 100$$

### 2.2. Animals and experimental design

Animal procedures were approved by our institutional ethical committee of the Vrije Universiteit Brussel (permit number: LA1230277) and performed in accordance with the national guidelines and regulations. Approval was obtained for this specific study (permit number: 16-277-2). Animals were housed in the university animal house according to the regulations of Belgian and EU legislation; food and water supply was given ad libitum. Animal pain and suffering was assessed as “moderate” by the ethical committee, requiring no special treatment. Male Balb/c mice, weighing approximately 25 g (n = 56), 9–11 weeks of age, were obtained from Charles River laboratories (Saint Germain Nuelles, France). In the first experiment, mice were randomly divided into 2 groups, untreated control group and STZ-treated group. Mice in the STZ-treated group were injected intraperitoneally with a single high dose of 200 mg/kg streptozotocin, dissolved in 0.1 M citrate buffered saline, to induce beta cell damage. Because plasma analyses required at least 300 μL sampling blood volume per mouse, blood collection was performed only once per animal. For this, STZ-treated mice were subdivided into 3 groups for blood collection at 4, 6 and 30 hours post-STZ administration. Tail vein blood glucose was measured using Glucocard Memory strips (GlucoMenLXPlus+, Menarini diagnostics, Zaventem, Belgium). Blood samples were collected via cardiac puncture during deep anesthesia with Isoflurane into tubes containing EDTA-aprotinin, and plasma was obtained by centrifugation. Mice were sacrificed immediately after blood collection by cervical dislocation. In the second set of experiments, mice received 10 mg/kg PPAG treatment or vehicle (0.9% NaCl) by oral gavage for four consecutive days. After two days of treatment, mice were injected with 200 mg/kg streptozotocin. Mice treated with the anti-diabetic exendin-4 (Sigma Aldrich), at a daily dose of 10 μg/kg via intraperitoneal injection, were used as positive control for beta cell protection. Body weight and glycemia were monitored everyday throughout the experiment. Blood samples were collected by cardiac puncture at 30 hours post-STZ injection and mice were subsequently euthanized by cervical dislocation during Isoflurane anesthesia.

### 2.3. Immunohistochemistry

Pancreata were fixed overnight in formaldehyde, dehydrated and embedded in paraffin. Four μm thin sections were cut, mounted on glass and stained. Pancreatic sections were stained with guinea pig anti-insulin polyclonal antibody (1:3000; Van Schravendijk, Brussels) and species-matched Cy3-conjugated secondary antibody (Jackson), and counterstained with Hoechst (Sigma Aldrich). Images were acquired with a Carl Zeiss multi-photon confocal laser scanning microscope LSM710 and analyzed using ImageJ and Volocity software.

### 2.4. MiR-375 measurement

MiR-375 was measured after sequence-specific capture by hybridization using Taqman miRNA ABC Purification Kit Human Panel A (Applied Biosystems, Waltham, Massachusetts). After the addition of ABC buffer, a dilution series of RNase free, HPLC purified RNA duplex microRNA 375 (active strand: 5PHOS/rUrUrUrGrUrUrCrGrUrUrCrGrGrCrUrCrGrCrGmUmGmA (manufacturing ID M118344864) and inactive strand: mAmArAmCrAmArGmCrAmArGmCrCmGrAmGrCmGrCrA (manufacturing ID M118344859) (Integrated DNA technologies, Coralville, Iowa)) was added to control matrix (plasma from Balb/c mice and DMEM medium supplemented with 10% FCS) for generation of a miR-375 standard curve. For reverse transcription (RT) (TaqMan MicroRNA Reverse Transcription Kit and TaqMan MicroRNA Assays (Applied Biosystems)), 5 μL RNA extract, 0.30 μL 100 mM dNTP mix, 3.00 μL Multiscribe Reverse Transcriptase, 1.50 μL 10x Reverse Transcription Buffer, 0.20 μL RNase Inhibitor and 5.00 μL RT primer pool were used. For the primer pool, 6 μL RT primer was added in a total volume of 500 μL TE buffer (Ambion, Waltham, Massachusetts). RT product was 6.7 times diluted and subsequently analyzed (with 9 μL of diluted RT product, 1 μL Taqman primer and 10 μL TaqMan Universal Master Mix II, no UNG) on a 7900HT Fast Real-Time PCR System using the default thermal-cycling conditions (Applied Biosystems).

### 2.5. Beta cell mass

Beta cell mass was estimated by quantifying total number of beta cells within a pancreatic section area of at least 100 mm^2^ for each mouse and expressed as the number of beta cells per area of pancreatic tissue (cells/mm^2^).

### 2.6. Apoptosis

Beta cell apoptosis was visualized by TUNEL staining of pancreatic sections using the In Situ Cell Death Detection kit (Roche, Brussels, Belgium). Level of beta cell apoptosis was quantified by dividing the number of TUNEL-positive beta cells by the total counted beta cell cells.

### 2.7. Statistics

Data were analyzed with GraphPad Prism using Linear Regression analysis, Mann-Whitney U test, 1- and 2-way ANOVA with Bonferonni post hoc test and Kruskal-Wallis test with Dunn’s Multiple Comparison test. P-value below 0.05 was considered as statistically significant. All values are presented as mean ± SEM.

## 3. Results

### 3.1. Glycemia and miR-375 release following STZ

The aim of this study was to determine whether miR-375 can be used as a biomarker to assess the beta cytoprotective effect of antidiabetic drugs. We used a short-term in vivo model of acute damage wherein mice are treated with a single high dose of STZ. We previously reported that in this model orally administered PPAG was able to delay the onset of hyperglycemia and to protect beta cells against acute cell death caused by STZ. The beta cells could not be protected from the long-term genotoxic effect of STZ and therefore this model can only be used to assess short-term protective effects with 30 hours post-STZ as the most interesting time point [12].

**Fig 1:**
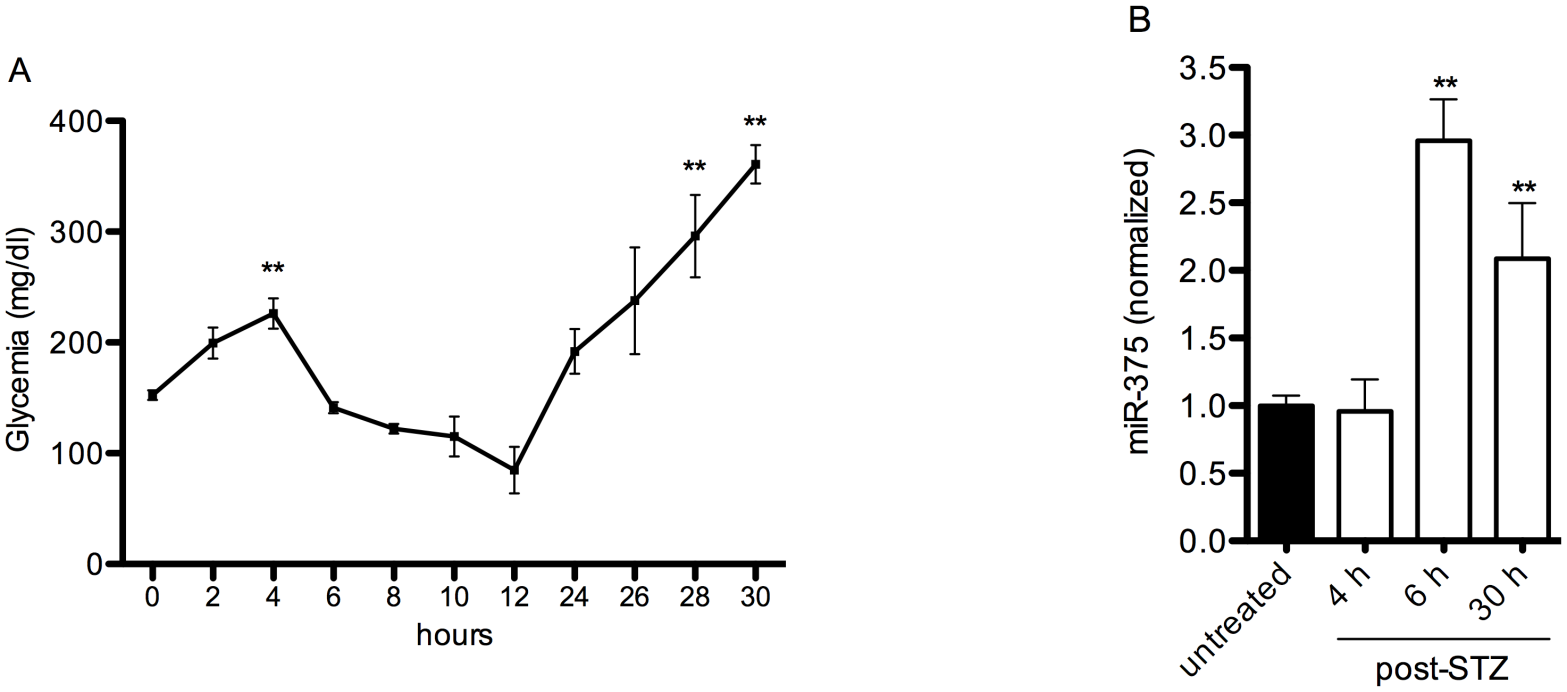
Blood glycemia and miR-375 blood levels after STZ injection. Blood glucose levels were measured at several time points after STZ injection (A). For time points 2, 8, 10, 12, 26 and 28 hours N = 4; For time points 0, 4, 6, 24 and 30 hours N = 21. ** p<0.01 versus time point 0 hour. MiR-375 plasma levels (B) were measured in untreated mice (N = 6) and 4 (N = 3), 6 (N = 4) and 30 hours (N = 4) after injection of STZ. ** p<0.01 versus untreated mice.

Blood glucose levels were increased after STZ administration following the characteristic biphasic pattern [20], with an increase at 4 hours and at 24–30 hours (Fig 1A).

In a first set of experiments plasma levels of miR-375 were measured in untreated control mice and after 4, 6 or 30 hours after STZ administration. MiR-375 in the blood plasma was detectable in all mice and remained stable at baseline level until 4 hours after STZ. Baseline levels ranged between 0.21 and 1.13 pmol/L (S1 Fig). A significant, threefold increase in miR-375 level was observed at 6 hours and remained high 30 hours after STZ (Fig 1B).

### 3.2. In vitro release of miR-375 release following STZ

**Fig 2:**
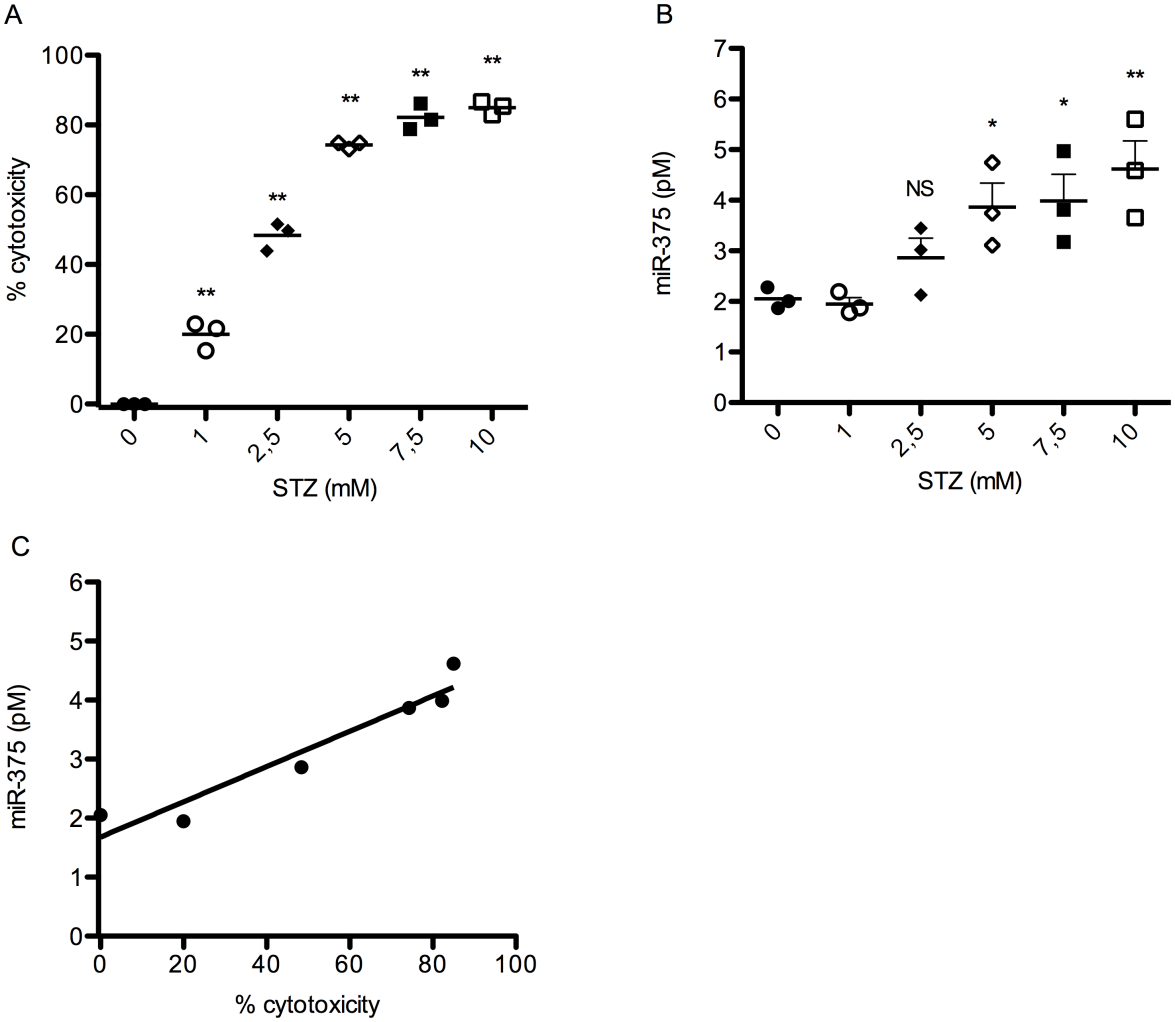
Correlation between cytotoxicity assay and miR-375 release in culture supernatant of MIN-6 cells. MIN-6 cells were incubated with different concentration of streptozotocin for 18 hours. Culture supernatant was collected and cytotoxicity assay (A) was performed on the remaining cells. Values are mean +/- SEM from three independent experiments performed in triplicate. **p<0.01 versus 0 mM STZ. MiR-375 analysis (B) was performed to evaluate miR-375 discharge. Values are mean +/- SEM from three independent experiments performed in triplicate. NS not significant; *p<0.05; **p<0.01 versus 0 mM STZ. Correlation between cytotoxicity and miR-375 release was calculated (C).

We also analyzed in vitro miR-375 release by the mouse beta cell line MIN-6 after an 18-hour exposure of the cells to various concentrations of STZ in the culture medium (range 0–10 mM). An overt cytotoxic effect of STZ became apparent at a STZ-concentration equal to or higher than 2.5 mM (Fig 2A). Increased release of miR-375 in the medium appeared at 2.5 mM STZ but not at lower concentrations (Fig 2B). Approximately a doubling of the baseline miR-375 concentration was observed at 5 mM STZ where nearly 80% of cell loss occurred. There was an excellent correlation between cytotoxicity/cell loss and miR-375 release, with a correlation coefficient R = 0.958 after linear regression (Fig 2C).

### 3.3. Beta cell protection by PPAG and exendin-4

**Fig 3:**
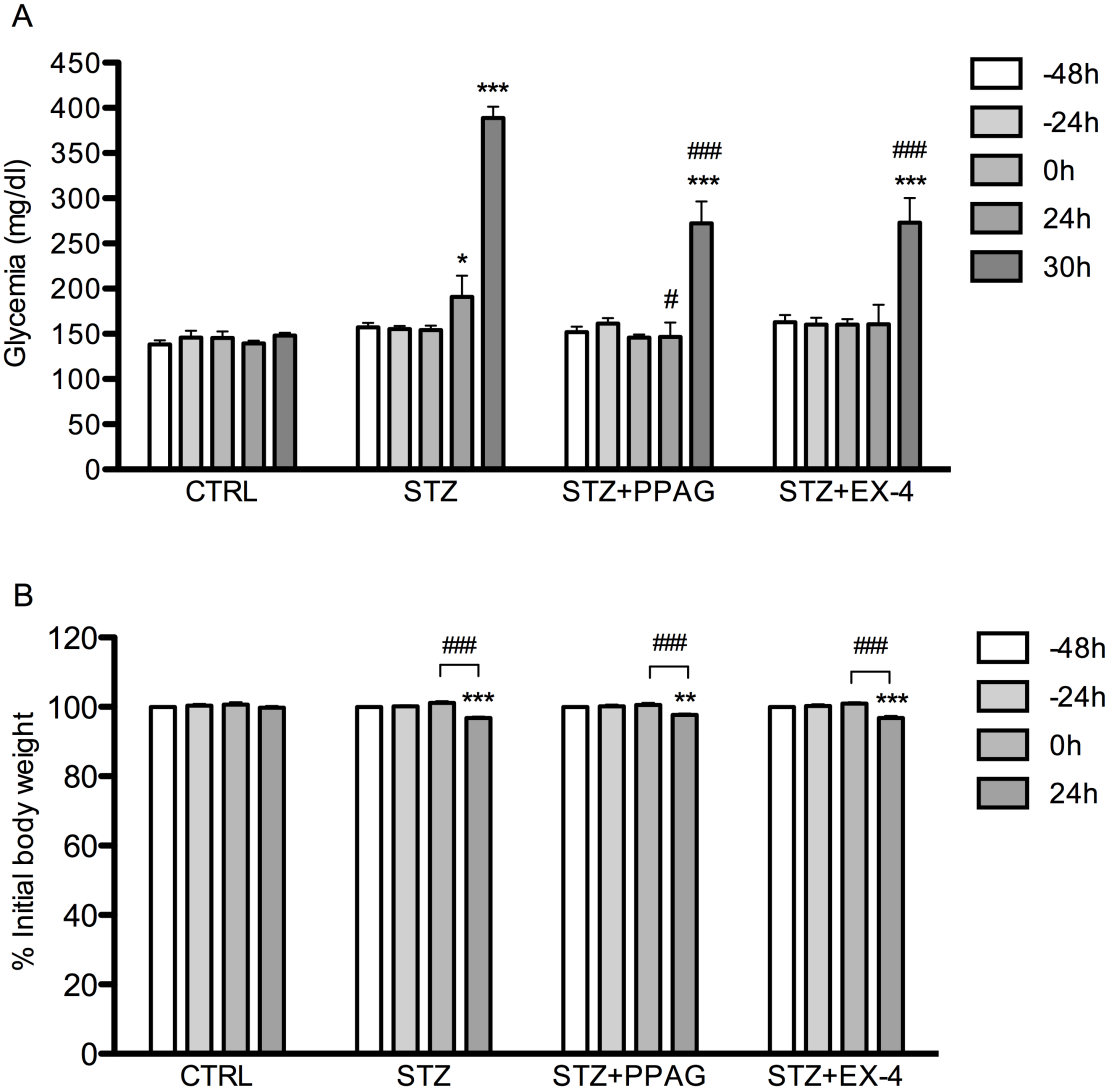
Glycemia and body weight in mice treated with STZ, PPAG or exendin-4. Mice were treated with vehicle (CTRL), PPAG or exendin-4 (EX-4) at -48, -24, 0 and 24 hours. One injection of STZ or vehicle was given at time point 0 hour. Non-fasting glycemia (A) was measured at indicated time points (N = 10). *p<0.05; ***p<0.001 compared to CTRL. #p<0.05; ###p<0.001 compared to STZ. Body weight (% initial -48h weight) was measured (B) at indicated time points (N = 10). **p<0.01; ***p<0.001 compared to CTRL. ##p<0.01; ###p<0.001 Effect of 24 hours STZ.

**Fig 4:**
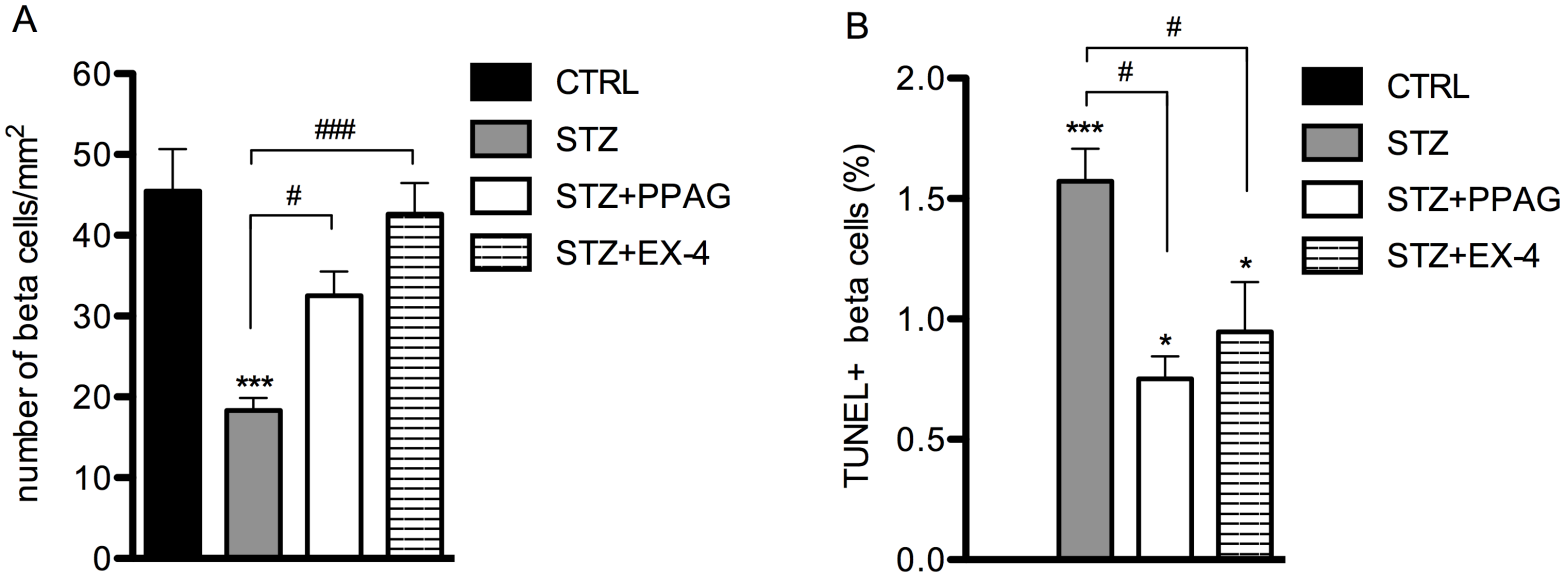
Beta cell mass and apoptosis in mice treated with STZ alone, STZ + PPAG or STZ + exendin-4. Mice were treated with vehicle (CTRL; N = 5), PPAG (N = 5) or exendin-4 (EX-4; N = 4) at -48, -24, 0 and 24 hours. One injection of STZ (N = 13) or vehicle (N = 5) was given at time point 0 hour. Thirty hours after STZ injection, beta cell mass (A) was measured. ***p<0.001 compared to CTRL mice. #p<0.05; ###p<0.001 Effect of PPAG and exendin-4 on STZ-induced decrease of beta cell mass. Beta cell apoptosis (B) was determined with TUNEL staining. *p<0.05; ***p<0.001 compared to CTRL mice. #p<0.05 Effect of PPAG and exendin-4 on STZ-induced increase of beta cell apoptosis.

In another set of experiments, mice were treated with either PPAG or exendin-4 (EX-4) to evaluate the protective effect against STZ-damage in vivo after 30 hours. Treatment with PPAG or exendin-4 significantly attenuated the STZ-induced hyperglycemia (Fig 3A). Body weight was not affected by the treatments, except that STZ treatment slightly lowered body weight in all groups of animals (Fig 3B). To examine the beta cell protective effect of PPAG or exendin-4, beta cell mass and beta cell apoptosis were analyzed. After STZ treatment, beta cell mass, measured as the number of insulin-positive cells over total tissue area, showed a decrease of about 60% at 30 hours post-STZ. The loss of beta cell mass was significantly attenuated by both PPAG and exendin-4 (Fig 4A). The frequency of apoptotic beta cells was determined by counting the percentage of insulin-positive cells that were also positive for TUNEL-staining. Whereas apoptotic beta cells were virtually absent in untreated control mice, they were present in STZ-treated mice. In line with the observations on loss of beta cell mass, the frequency of beta cell apoptosis was significantly reduced by treatment with PPAG or exendin-4 (Fig 4B).

**Fig 5:**
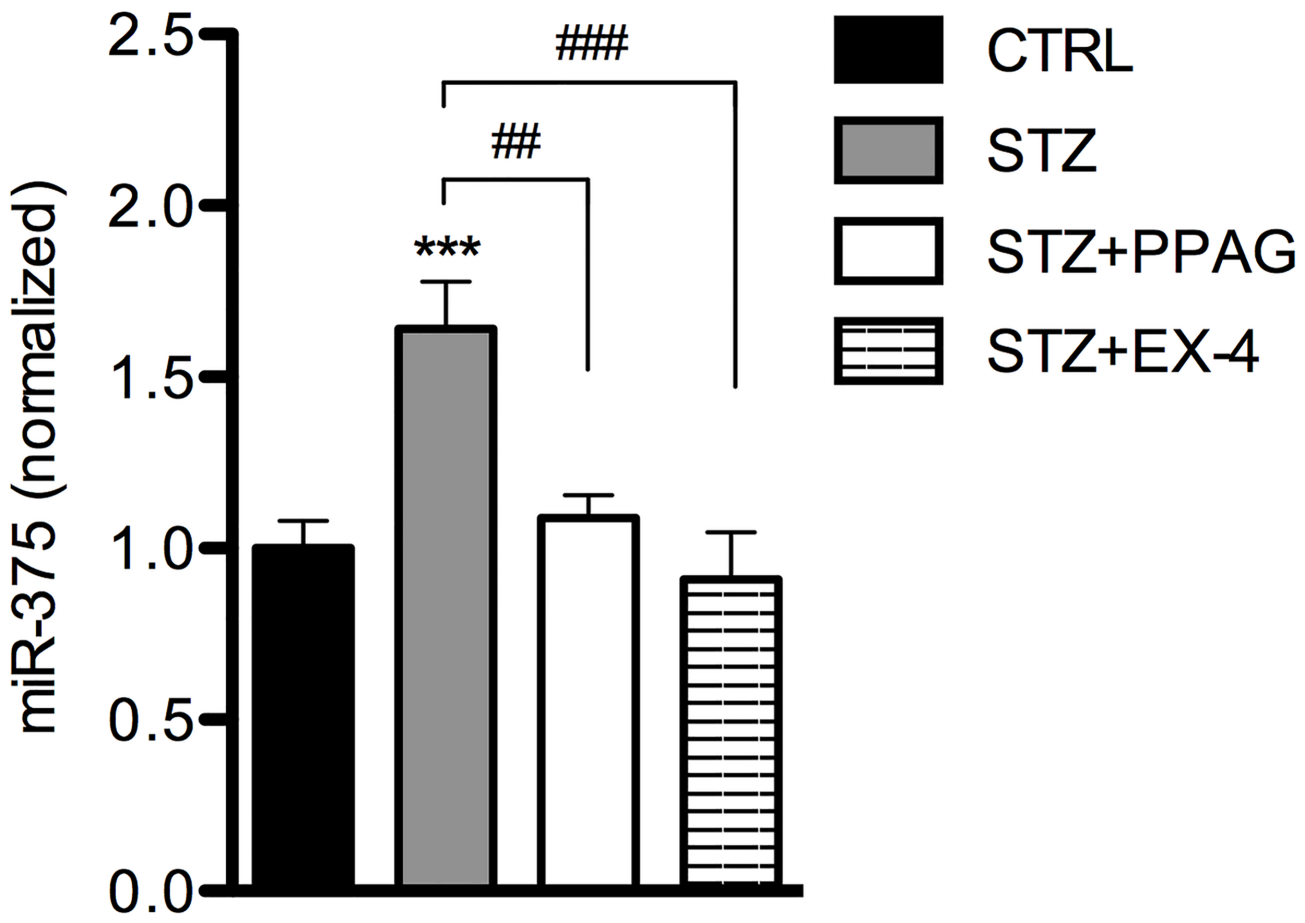
MiR-375 in blood plasma of mice treated with STZ, STZ + PPAG or STZ + exendin-4. Mice were treated with vehicle (CTRL; N = 15), PPAG (N = 12) or exendin-4 (EX-4; N = 8) at -48, -24, 0 and 24 hours. One injection of STZ (N = 35) or vehicle (N = 15) was given at time point 0 hour. Thirty hours after STZ injection, blood was collected and miR-375 was measured. ***p<0.001 compared to CTRL mice. ##p<0.01; ###p<0.001 Effect of PPAG and exendin-4 on STZ-induced increase of miR-375 levels.

To evaluate whether the beta cytoprotective effect of PPAG and exendin-4 can be assessed by circulating miR-375, plasma levels of miR-375 were measured. Mice that were treated with PPAG or exendin-4 showed no increase in plasma miR-375 (Fig 5). Thus, PPAG and exendin-4 prevented the release of miR-375 in the blood.

These results confirm that PPAG and exendin-4 can protect beta cells against STZ-induced cell death and cell loss. Furthermore, a correlation was found between miR-375 and beta cell death or survival, indicating that circulating miR-375 can be used as a biomarker to predict these effects.

## 4. Discussion

This study provides evidence that miR-375 can be used as a serological biomarker to detect beta cell death and to test drugs that could protect beta cells against cell death. It has previously been reported that circulating miR-375 levels can serve as a tool to predict diabetes in STZ-treated mice [17]. However, in their study no direct link was examined between circulating miR-375 levels and beta cell mass or beta cell death in vivo.

In our study, the increase in blood miR-375 level 30 hours after STZ administration was nearly twofold compared to controls. In these conditions the beta cell mass was reduced by approximately 60%. In vitro, similar changes were observed on a beta cell line with nearly a doubling of the miR-375 concentration released in the culture supernatant following STZ exposure, coinciding with an approximate 80% beta cell loss.

Treatment with the beta cytoprotective compounds PPAG and exendin-4 significantly reduced the STZ-induced beta cell mass depletion in vivo. Both compounds also decreased the frequency of apoptotic beta cells in pancreatic tissue. Furthermore, the cytoprotective effect of PPAG and exendin-4 resulted into a normal level of circulating miR-375. While the numbers of TUNEL-positive (apoptotic) beta cells after STZ are significantly reduced by both PPAG or exendin-4 treatment, they are still higher than in control mice. This may be explained by the fact that STZ also induces necrotic beta cell death [12] and that miR-375 is mainly reflecting this type of cell death rather than apoptosis. The TUNEL method is a classic cytochemical application to detect DNA fragmentation and is generally considered the gold standard, and only available method, for apoptosis detection in tissue sections. However, it has been argued that in some cases TUNEL positivity might also result from necrotic cell death. Unfortunately, there exists at present no method to detect necrotic cell death in tissue sections.

Our data confirm that circulating miR-375 can be used as a biomarker to detect damage to the pancreatic beta cell mass. In addition, we demonstrate that the beta cytoprotective effect of (experimental) antidiabetic drugs like PPAG or exendin-4 can be assessed by measuring circulating miR-375 levels. To our knowledge, this is the first description of the use of miRNA for in vivo beta cytoprotective drug testing.

STZ treatment may not be very relevant to clinical diabetes and it therefore remains necessary to investigate this in other models of diabetes. In rodents, STZ induces both necrotic and apoptotic beta cell death [12,20]. Apoptosis is the major cell death mechanism induced by STZ in beta cells cultured at high glucose concentrations in vitro [12] and is also considered as the main mechanism of beta cell death involved in diabetic conditions in humans [1–4]. It is possible that miRNAs are mainly released from necrotic cells since their plasma membrane becomes permeable, and less from apoptotic cells as the apoptotic process does not directly involve permabilization of the plasma membrane. However, apoptosis progresses to secondary necrosis if apoptotic cells are not efficiently removed by macrophages [21]. A major question is whether the extent of beta cell death under pre-diabetic or diabetic conditions in humans will be sufficient to be serologically traceable by miRNA analysis. Interestingly, the expression levels of diabetes-related miRNAs, including miR-375, in serum were found to be significantly elevated in type 2 diabetes patients compared with pre-diabetic and normal glucose-tolerant individuals [22,23]. Also higher miR-375 levels could be detected in the circulation of type 1 diabetes subjects [19].

For unknown reasons, mice show higher baseline levels of miR-375 in blood than humans, suggesting contribution to the circulating miR-375 pool from other tissues. As a consequence, minor fluctuations in baseline levels will mask events of low grade or asynchronous beta cell death in mice, decreasing the biomarker’s sensitivity and specificity in less extreme models of beta cell toxicity. In humans, the lower baseline levels and their lower intra- and inter-individual variations, favour the biomarker’s diagnostic performance, allowing its use to detect beta cell apoptosis up to weeks after beta cell transplantation, or around the clinical onset phase of type 1 diabetes (S. Roels, manuscript in preparation). A second limitation in mice, is the relatively large volume of blood that must be sampled (20% of total blood volume) making it impossible to repeatedly sample from the same mouse. Future experimental work must be directed at down-scaling the protocol so as to be able to measure miRNAs in capillary sampled blood, without affecting assay sensitivity.

Our study confirms the beta cytoprotective effect of PPAG that we previously reported in mice treated with a chronic diabetogenic diet [10] or an acute STZ-injury [12]. In addition, we now show that the protective efficacy of PPAG is comparable to that of exendin-4, an established antidiabetic drug with demonstrated beta cytoprotective effects [24–26]. However, whereas exendin-4 and its peptide analogs must be administered by injections, PPAG is administered orally making it an interesting drug candidate.

So far, betacytoprotective effects in vivo need to be assessed by cumbersome morphometric measurements in pancreatic tissue samples. Our results indicate that microRNA measurements in plasma provide an additional method to test potential cytoprotective drug candidates.

## Supporting information

S1 Fig Baseline levels of serum miRNA-375 in Balb/c mice. Average +/- SEM of baseline levels of serum miRNA-375 of 15 untreated Balb/c mice.
(TIF)
